# Characteristics of clinical trials of influenza and respiratory syncytial virus registered in ClinicalTrials.gov between 2014 and 2021

**DOI:** 10.3389/fpubh.2023.1171975

**Published:** 2023-09-28

**Authors:** David Lora, Ana García-Reyne, Antonio Lalueza, Guillermo Maestro de la Calle, María Ruíz-Ruigómez, Enrique J. Calderón, Miguel Menéndez-Orenga

**Affiliations:** ^1^Instituto de Investigación Sanitaria del Hospital Universitario 12 de Octubre (imas12), Madrid, Spain; ^2^Spanish Clinical Research Network (SCReN), Madrid, Spain; ^3^Facultad de Estudios Estadísticos, Universidad Complutense de Madrid (UCM), Madrid, Spain; ^4^Servicio de Medicina Interna, Hospital Universitario 12 de Octubre, Madrid, Spain; ^5^Hospital Universitario 12 de Octubre, Madrid, Spain; ^6^Facultad de Medicina, Universidad Complutense de Madrid (UCM), Madrid, Spain; ^7^CIBER de Enfermedades Infecciosas (CIBERINFEC), Madrid, Spain; ^8^Servicio de Medicina Interna, Antimicrobial Stewardship Program, Hospital Universitario 12 de Octubre, Madrid, Spain; ^9^Instituto de Biomedicina de Sevilla, Hospital Universitario Virgen del Rocío, Consejo Superior de Investigaciones Científicas, Universidad de Sevilla, Sevilla, Spain; ^10^Centro de Investigación Biomédica en Red de Epidemiología y Salud Pública (CIBERESP), Madrid, Spain; ^11^Departamento de Medicina, Facultad de Medicina, Universidad de Sevilla, Sevilla, Spain; ^12^Servicio Madrileño de Salud, Centro de Salud La Ventilla, Madrid, Spain

**Keywords:** influenza virus, respiratory syncytial virus (RSV), infectious diseases, clinical trials registry, randomized clinical trial (RCT)

## Abstract

The randomized clinical trial (RCT) is the ideal and mandatory type of study to verify the effect and safety of a drug. Our aim is to examine the fundamental characteristics of interventional clinical trials on influenza and respiratory syncytial virus (RSV). This is a cross-sectional study of RCTs on influenza and RSV in humans between 2014 and 2021 registered in ClinicalTrials.gov. A total of 516 studies were identified: 94 for RSV, 423 for influenza, and 1 for both viruses. There were 51 RCTs of RSV vaccines (54.3%) and 344 (81.3%) for influenza virus vaccines (*p* < 0.001). Twelve (12.8%) RCTs for RSV were conducted only with women, and 6 were conducted only with pregnant women; for RCTs for influenza, 4 (0.9%) and 3, respectively. For RSV, 29 (31%) of the RCTs were exclusive to people under 5 years of age, and 21 (5%) for influenza virus (*p* < 0.001). For RSV, there are no RCTs exclusively for people older than or equal to 65 years and no phase 4 trials. RCTs on influenza virus and RSV has focused on vaccines. For the influenza virus, research has been consolidated, and for RSV, research is still in the development phase and directed at children and pregnant women.

## Graphical abstract

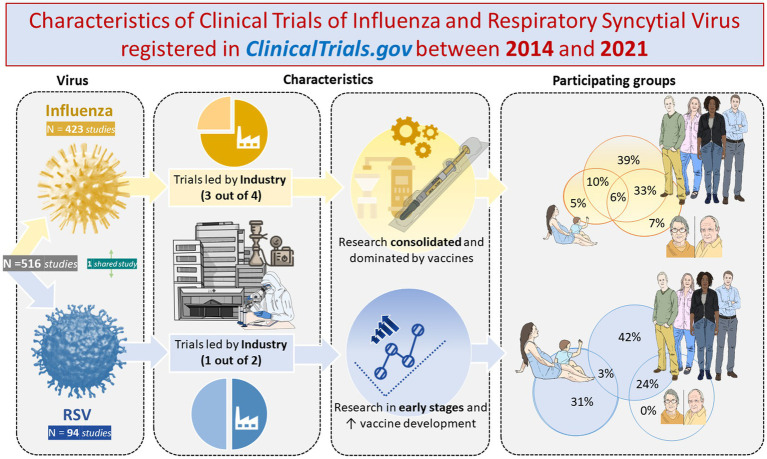

## Introduction

Respiratory syncytial virus (RSV) and influenza viruses are important causes of morbidity and mortality globally (1, 2). Both respiratory viral infections can result in severe disease and death in older adult individuals, children, pregnant women and people with underlying chronic conditions, even more so in low- and middle-income countries (1–4). There is an urgent need for better tools to prevent, detect, control and treat influenza and RSV, including more effective vaccines and antiviral drugs for influenza (4–6) and obtaining vaccines licensed for RSV (7–9).

Clinical trials with drugs are the ideal and mandatory type of study to verify the effect and safety of a drug. The registration of the clinical trial protocol is mandatory for the promoters, who must record the key elements and report the results of the clinical trial and adverse events (10) regardless of the direction or strength of the results (11). The transparency of information and public access to the results of clinical trials are essential for the protection and promotion of public health. In this way, scientific knowledge and its dissemination among health professionals and citizens are promoted, progress in clinical research is promoted, and value is given to the participation of patients in clinical trials for the benefit of all.

Different countries and organizations have specific regulations and their own registries for clinical trials. ClinicalTrials.gov is a Web-based resource that provides patients, their family members, health care professionals, researchers, and the public with easy access to information on publicly and privately supported clinical studies on a wide range of diseases and conditions conducted in the United States (12). There are different works that analyze the clinical trials registered in ClinicalTrials.gov in a general way (10), as well as for infectious diseases (9, 13) and the older adult population (14) or for SARS-CoV-2 (15–17). There is no specific one for the flu.

The present work aims to evaluate the clinical trials registered in ClinicalTrials.gov on influenza and RSV, the two most prevalent respiratory viral diseases before the emergence of COVID-19, between 2014 and 2021.

## Materials and methods

Cross-sectional study of clinical trials on influenza or RSV in humans registered in ClinicalTrial.gov between 2014 and 2021. The data were obtained as of February 2023 through “the Database for Aggregate Analysis of ClinicalTrials.gov (AACT) (12, 18), as a cloud-hosted PostgreSQL database” using R’s RPostgreSQL library (19). ClinicalTrials.gov studies that met the following conditions were included: (1) In the data table of “studies,” those which were registered as “interventional” under the variable “study_type” and had the phase of the clinical trial defined under the variable “phase”; (2) Those that have a start date between 2014 and 2021, inclusive, under the “start_date” variable of the “studies” table; (3) Those that have the term Medical Subject Heading (MeSH) equal to “Influenza, Human” or “Respiratory Syncytial Virus Infections” under “browse_conditions” in the data table. Finally, the clinical trials obtained were manually reviewed, eliminating those that did not satisfy the previously established conditions or that were studies on vaccination policies.

The information extracted from the clinical trials was established under general characteristics and design, and methodological attributes. The following general characteristics were established: primary research proposal (treatment, prevention, diagnostic, and other), study registration established as the beginning of recruitment before or after registration in ClinicalTrials.gov, source of funding (Industry, National Institutes of Health [NIH] and Other), review by a DMC (Data Monitoring Committee), regions involved in the study (Africa, Asia and Pacific, Central and South America, Europe, Middle East, North America and Missing), study status, population included in relation to gender and age, and type of molecule evaluated in clinical trials (antiviral drugs, antibodies, vaccines and others). In the category other than the type of molecule, treatments such as “traditional Chinese medicine,” probiotics, antifungals, antiprotozoals, antibiotics and the like were included. The characteristics of the design and methodology collected were: documentation of the protocol and the statistical analysis plan (SAP), phase of clinical trial, allocation, interventional group, number of arms, number of recruited patients, and blinding. When possible, values of missing characteristics were inferred based on other available data. For example, for studies reporting an interventional model of a single group and number of groups as 1, the value of allocation and blinding was designated as nonrandomized and open, respectively (10).

All categorical variables were reported with absolute and relative frequencies. Data were stratified by influenza virus and RSV. Comparisons between groups were performed using the two-tailed chi-square test with an alpha error equal to 0.05. The graphic information was represented through Venn diagrams and ternary diagrams. The ternary diagram is a triangular graph that visualizes in a two-dimensional way the relationships between phase (represented by dots in the diagram) and the percentage of intervention/treatment (represented on each of the three axes). This graph was also used to represent the relationship between the years and the intervention/treatment. In this representation, the category other than the variable type of intervention molecule was eliminated. The Venn diagram presents the age groups to which clinical trials are directed. The age groups were established based on the scientific literature (3, 20): (1) less than 5 years old, (2) greater than and equal to 5 years old and less than 18 years old, (3) greater than or equal to 18 years old and less than 65 years old, and (4) greater than or equal to 65 years old. R was used for all statistical and graphical analyses (19) using the R libraries (packages) ggVennDiagram and Ternary.

## Results

A total of 516 clinical trials that met the established criteria were selected from the total of 441,919 records included in ClinicalTrials.gov. A total of 423 clinical trials were of influenza virus, and 94 were of RSV. A study is shared in both groups of viruses.

Table 1 shows the characteristics of the clinical trials. The most frequent primary endpoint in clinical trials was prevention, with 56 (60.2%) for RSV and 282 (67.0%) for influenza. The industry conducted 78 (83.0%) clinical trials for RSV versus 237 (56.0%) for influenza, *p* < 0.001. Significant differences were found between the percentage of vaccines developed for RSV, 51 (54.3%), versus the percentage of vaccines developed for influenza virus 344 (81.3%), with a *p* < 0.001. The pharmaceutical industry participated in more than 50% of the RSV and influenza clinical trials. In 49.2% of the clinical trials registered in ClinicalTrials.gov, there was participation of North American centers. Highlight the interest in RSV in developing clinical trials only in women, 12 (12.8%), 6 directed at pregnant women, compared to 4 (0.9%) clinical trials for women in the influenza virus, 3 directed at pregnant women.

**Table 1:** Characteristics of clinical trials on influenza and RSV registered in ClinicalTrials.gov between 2014 and 2021.

	Level	Overall (*n* = 516)	RSV (*n* = 94)	Influenza virus (*n* = 423)
Primary purpose	Diagnostic	2 (0.4)	0 (0.0)	2 (0.5)
	Prevention	337 (65.7)	56 (60.2)	282 (67.0)
	Treatment	121 (23.6)	29 (31.2)	92 (21.9)
	Other	53 (10.6)	8 (8.6)	45 (10.7)
Intervention/Treatment	Anticuerpos	28 (5.4)	10 (10.6)	18 (4.3)
	Antivirals	68 (13.2)	31 (33.0)	37 (8.7)
	Vacuna	394 (76.4)*	51 (54.3)	344 (81.3)
	Otros	26 (5.0)	2 (2.1)	24 (5.7)
Study registration	Before first participant enrolled	199 (38.6)	32 (34.0)	168 (39.7)
	After first participant enrolled	317 (61.4)	62 (66.0)	255 (60.3)
Lead sponsor	Industry	314 (60.9)	78 (83.0)	237 (56.0)
	NIH	54 (10.5)	14 (14.9)	40 (9.5)
	Other	148 (28.7)	2 (2.1)	146 (34.5)
DMC	Study has DMC	250 (56.7)	37 (43.5)	214 (59.9)
	Study does not have DMC	191 (43.3)	48 (56.5)	143 (40.1)
Region**	Africa	36 (7.0)	17 (18.1)	19 (4.5)
	Asia And Pacific	165 (32.0)	41 (43.6)	125 (29.6)
	Central and South America	48 (9.3)	22 (23.4)	26 (6.1)
	Europe	154 (29.8)	49 (52.1)	106 (25.1)
	Middle East	31 (6.0)	13 (13.8)	18 (4.3)
	North America	254 (49.2)	61 (64.9)	194 (45.9)
	Missing	35 (6.8)	2 (2.1)	33 (7.8)
Overall status	Active, not recruiting	29 (5.6)	9 (9.6)	20 (4.7)
	Completed	388 (75.2)	69 (73.4)	320 (75.7)
	Enrolling by invitation	2 (0.4)	0 (0.0)	2 (0.5)
	Not yet recruiting	1 (0.2)	0 (0.0)	1 (0.2)
	Recruiting	30 (5.8)	4 (4.3)	26 (6.1)
	Suspended	2 (0.4)	1 (1.1)	1 (0.2)
	Terminated	21 (4.1)	7 (7.4)	14 (3.3)
	Unknown status	35 (6.8)	1 (1.1)	34 (8.0)
	Withdrawn	8 (1.6)	3 (3.2)	5 (1.2)
Gender	Both	496 (96.1)	80 (85.1)	416 (98.3)
	Female only	15 (2.9)	12 (12.8)	4 (0.9)
	Male only	5 (1.0)	2 (2.1)	3 (0.7)
Includes children (<18 years)	Yes	155 (30.0)	35 (37.2)	120 (28.4)
Includes older adult (≥65 years)	Yes	217 (42.1)	23 (24.5)	194 (45.9)
Dirigidos a mujeres embarazadas	Yes	9 (1.7)	6 (6.4)	3 (0.1)

RSV, Respiratory Syncytial Virus; DMC, Data Monitoring Committee; NIH, National Institutes of Health. *Elimination of duplication of the coincident study of influenza and RSV. **Clinical trials can take place in more than one country. All cells show the absolute number of studies and the percentage of the total, *n* (%).

**Figure 1:**
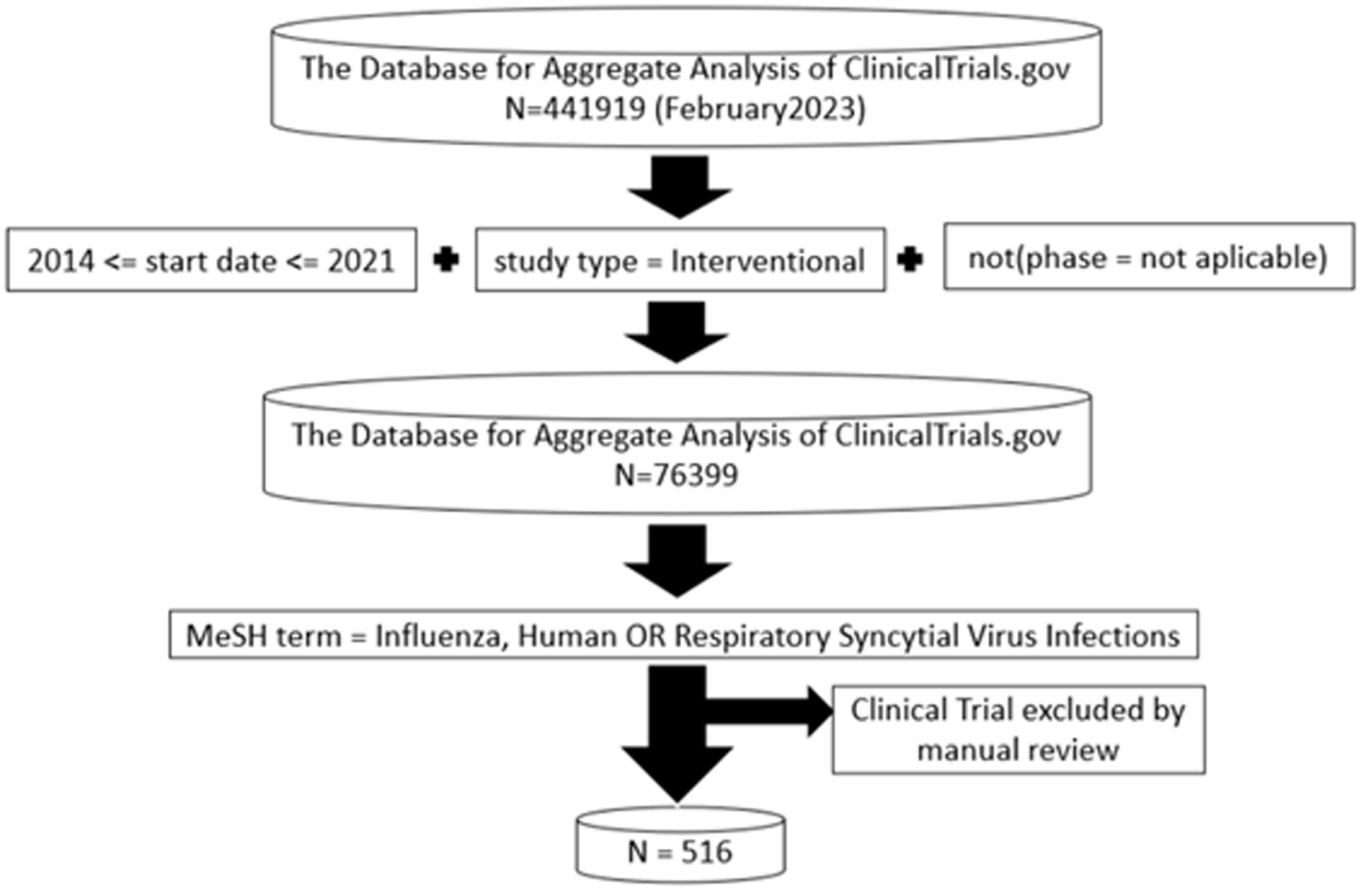
Trials dataset. Flow diagram depicting the derivation of the influenza virus and Respiratory Syncytal Virus.

The Venn diagram (Figure 1) shows the age composition of the clinical trials included in the study. We found that a significant percentage of RSV studies included patients under 5 years of age compared to influenza studies, 29 (31%) for RSV versus 21 (5%) for influenza studies, *p* < 0.001. On the other hand, 23 (24%) of the RSV clinical trials included a population aged 65 years or over, compared to 184 (46%) of the influenza trials, *p* < 0.001. It should be noted that no RSV study exclusively included patients aged 65 or over compared to 29 (7%) influenza studies.

Phase designs less than or equal to 2 are the majority in RSV, above 80%. On the other hand, in the influenza virus, it was observed that the investigation is distributed homogeneously in each of the phases. The usual allocation for clinical trials in both viruses was in parallel arms, greater than 80%, and with two arms, greater than 40% (Table 2). The number of clinical trials that published the protocol and the SAP was similar between both infectious diseases, 122 (23.8%).

**Table 2:** Design and methodology of clinical trials on influenza and RSV registered in ClinicalTrials.gov between 2014 and 2021.

	Level	Overall (*n* = 516)	RSV (*n* = 94)	Influenza virus (*n* = 423)
Phase	Early Phase 1	2 (0.4)	0 (0.0)	2 (0.5)
	Phase 1	129 (25.0)	41 (43.6)	88 (20.8)
	Phase 1/Phase 2	27 (5.2)	5 (5.3)	22 (5.2)
	Phase 2	125 (24.3)	33 (35.1)	92 (21.7)
	Phase 2/Phase 3	12 (2.3)	2 (2.1)	10 (2.4)
	Phase 3	113 (21.9)	13 (13.8)	101 (23.9)
	Phase 4	108 (20.9)	0 (0.0)	108 (25.5)
Allocation	Non-randomized	92 (17.8)	12 (12.8)	80 (18.9)
	Randomized	424 (82.2)	82 (87.2)	343 (81.1)
Assignment	Crossover	3 (0.6)	0 (0.0)	3 (0.7)
	Factorial	5 (1.0)	0 (0.0)	5 (1.2)
	Parallel	432 (83.9)	81 (86.2)	352 (83.4)
	Sequential	19 (3.7)	4 (4.3)	15 (3.6)
	Single Group	56 (10.9)	9 (9.6)	47 (11.2)
Number of arms	1	47 (9.1)	5 (5.3)	42 (9.9)
	2	223 (43.2)	45 (47.9)	178 (42.1)
	3	97 (18.8)	12 (12.8)	85 (20.1)
	4	65 (12.6)	13 (13.8)	52 (12.3)
	5	26 (5.0)	5 (5.3)	21 (5.0)
	6	28 (5.4)	7 (7.4)	21 (5.0)
	+ de 6	30 (5.8)	7 (7.4)	24 (5.7)
Enrollment	1–100	175 (34.4)	54 (59.3)	121 (28.9)
	100–1,000	253 (49.8)	26 (28.6)	227 (54.3)
	>1,000	80 (15.7)	11 (12.1)	70 (16.7)
Blinding	None (Open Label)	150 (29.1)	15 (16.0)	135 (32.0)
	Single	34 (6.6)	5 (5.3)	29 (6.9)
	Double	109 (21.2)	21 (22.3)	88 (20.9)
	Triple	64 (12.4)	18 (19.1)	46 (10.9)
	Quadruple	158 (30.7)	35 (37.2)	124 (29.4)
Study Protocol available	Yes	122 (23.8)	25 (25.6)	98 (23.2)
SAP available	Yes	122 (23.8)	25 (25.6)	98 (23.2)
Publicado en pubmed (February 2023)	Yes	139 (26.9)	23 (24.5)	116 (27.4)

RSV, Respiratory Syncytial Virus; SAP, Statistical Analysis Plan. All cells show the absolute number of studies and the percentage of the total, n (%).

**Figure 2:**
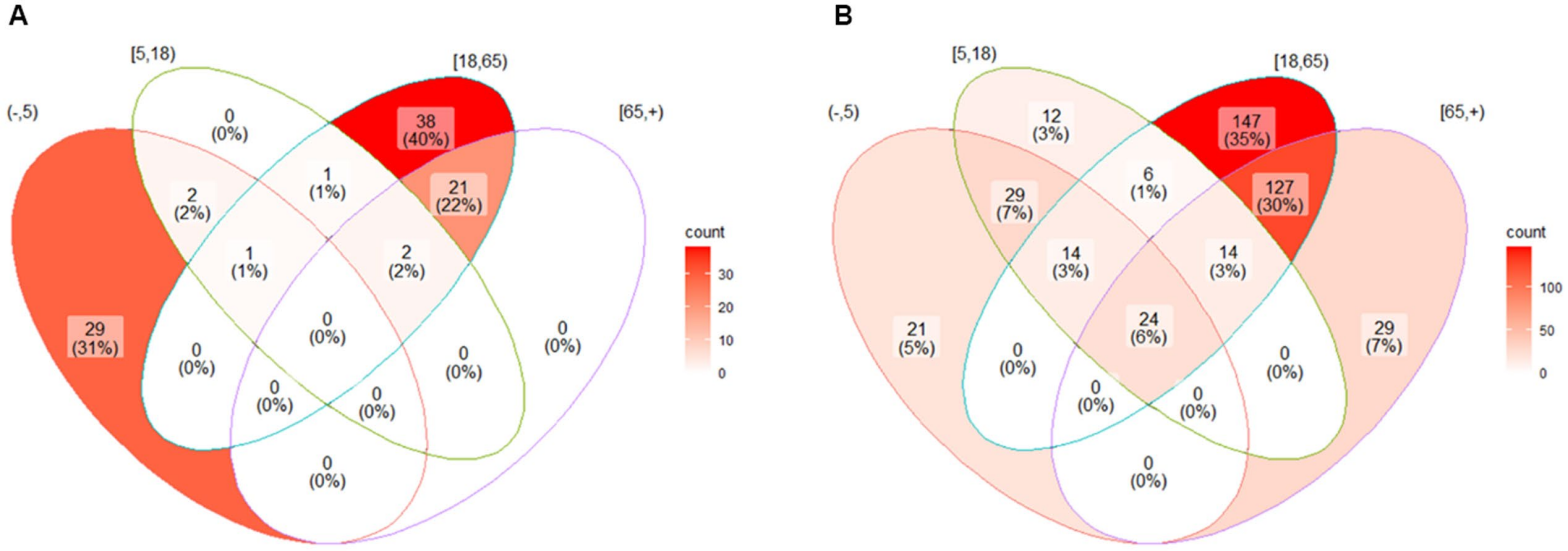
Venn diagram. The circles show the age strata where the clinical trials took place. The intersection of the circles shows the clinical trials with mixed age groups. The age groups considered are (1) less than 5 years old, (2) greater than and equal to 5 years old and less than 18 years old, (3) greater than or equal to 18 years old and less than 65 years old, and (4) greater than or equal to 65 years. The number of clinical trials and their percentage with respect to the total are presented for each of the strata.

Regarding the type of molecule investigated in the different phases of RSV clinical trials (Figure 2A), in phase 1 clinical trials, approximately 60% were vaccines, 33% antibodies and 5% antivirals. All phases (Figure 2A) accounted for between 45 and 70% of vaccine research, with the exception of phase 2/phase 3, in which 100% were antiviral, although there were only 2 studies. Figure 2B shows the evolution of the trials over the years included, noting that the vaccine trials have gone from 30% in 2014 to 68.8% in 2021. The number of RSV studies has remained stable throughout the years, ranging from 10 (2015, 2019) to 16 annual trials (2021).

**Figure 3:**
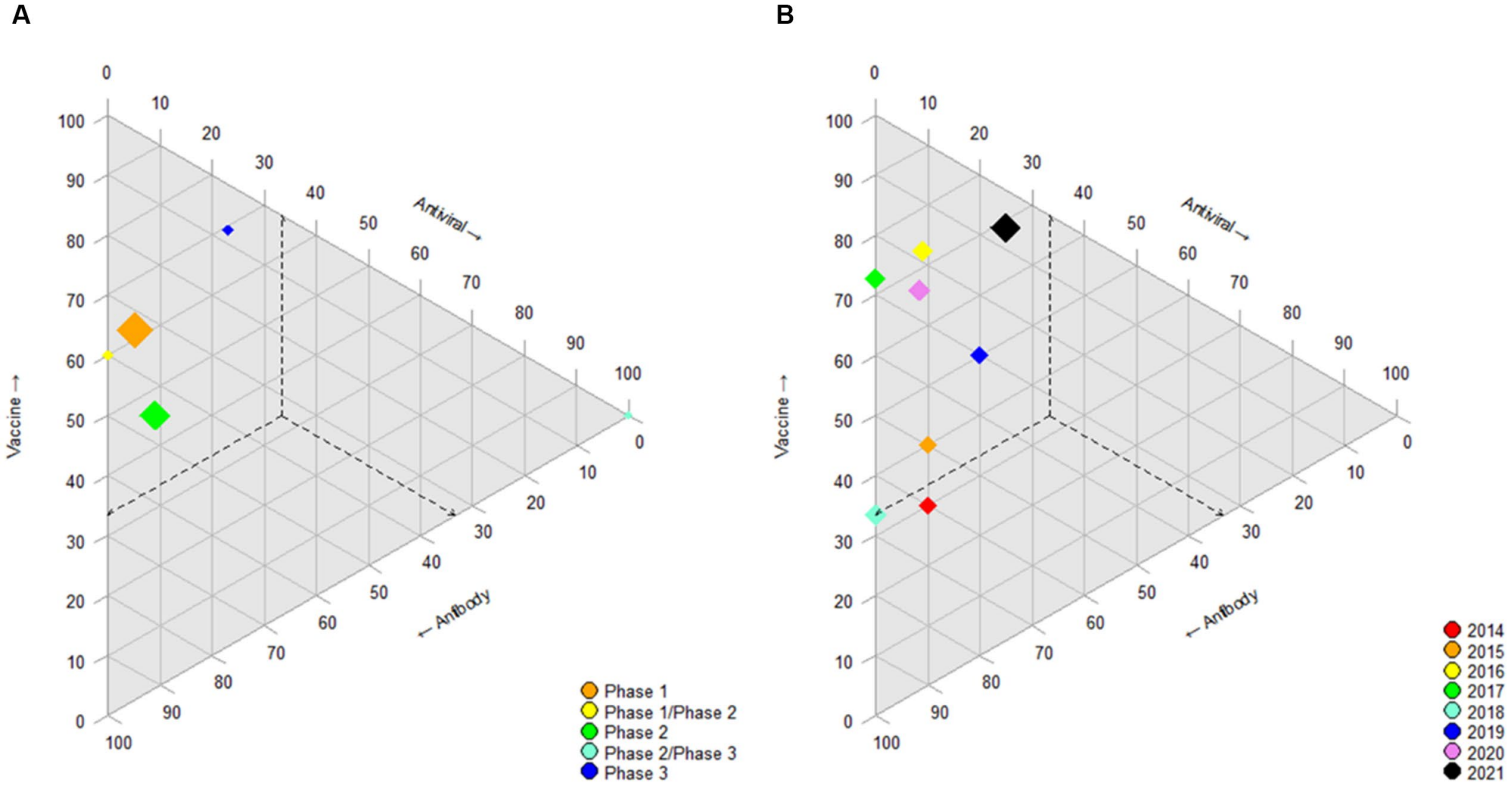
Ternary diagram. Ternary diagram representing the phase of clinical trial (or year) in accordance with their positions on each of the three axes for influenza virus. Each axis represents the percentage of intervention/treatment groups vaccine, antiviral and antibody. Other interventions or treatments are not shown. The dashed lines indicate the coordinates of the different phase (or year) leading to the point where intervention is located (as an example for interpretation). The size of the diamond represents the absolute number of trials in that category: a larger diamond size indicates a greater number of studies. **(A)** RSV-phase. **(B)** RSV year.

In the case of influenza (Figure 3A), more than 75% of the trials, regardless of the phase, are for vaccines, and in all years, more than 80%. In 2020 and 2021 (Figure 3B), the study of monoclonal antibodies against the influenza virus increased by up to 10%. The number of studies on the influenza virus decreased over the 8 years studied, from 67 clinical trials in 2014 to 46 by 2021 and 28 studies in 2020.

## Discussion

The data from our study show research mostly conducted by the pharmaceutical industry, with approximately half of the studies reporting the presence of DMC. For influenza, research is consolidated and dominated by the development of vaccines, compared to research on RSV in which studies in early stages abound and with less predominance of research on vaccines, although increasing. Research on influenza has a higher participation of the older adult compared to RSV research, in which the participation of children and pregnant women was clearly higher.

Different authors have characterized the clinical trials that appear in ClinicalTrials.gov (9, 10, 13). Only 38.4% of clinical trials targeting infectious diseases registered on ClinicalTrials.gov between 2007 and 2010 (13) had a primary objective of prevention, compared to 65.5% of the clinical trials of RSV and influenza aimed at prevention existing in ClinicalTrials.gov between 2014 and 2021 in our study. The increase may be due to the exclusion criteria for clinical trials established in our study. On the other hand, it is reasonable to think that research efforts are aimed at prevention given the high burden of morbidity and mortality at the global level of infectious viral diseases, such as influenza and RSV, as opposed to bacterial diseases. This high morbidity and mortality due to RSV in the older adult population (20), and the research strategies promoted in 2015 by the World Health Organization (WHO) to provide guidance on clinical endpoints and development pathways for vaccine trials with a focus on considerations of low- and middle-income countries (7) and shown in different reviews on vaccines and monoclonal antibodies (8, 9), agrees with the high percentage of RSV vaccine research found over the years. However, no differences were found between countries according to income, as in other viral diseases (13). Similarly, the consolidated research of influenza clinical trials is consistent with the marked development of vaccines (4). This is reflected in the high percentage of clinical trials on phase 4 vaccines, unlike emerging infectious diseases such as COVID-19 (15–17).

We have not identified registered clinical trials on RSV aimed exclusively at the older adult population. In influenza, a minority of the studies addressed only older adult individuals. Approximately 1 in 4 studies on RSV and 1 in 2 on influenza included the older adult population in some way. The development of specific vaccines for this age group with a high risk of hospitalization could reduce the total burden of viral respiratory diseases in clinics and hospitals during the winter months (20). Vaccines may show less efficacy in the older adult population, in whom they may be more necessary. Therefore, specific studies that include combined treatments could be convenient.

The study presents the limitations of the sources from which the data are collected (10, 12). First, the clinical trials for RSV and influenza viruses listed on ClinicalTrials.gov are not all clinical trials developed for those two viruses. However, all clinical trials developed in the United States must be registered in ClinicalTrial.gov; in addition, some journals, in order to publish the results of clinical trials, require registration in ClinicalTrials.gov within their editorial policy. Some authors (13) have suggested that after manual review, approximately 80% of clinical trials registered in The International Clinical Trials Registry Platform (ICTRP) are also registered in ClinicalTrials.gov. Second, the information registered in ClinicalTrials.gov is entered manually by the research team of the clinical trial and is susceptible to errors and presents missing data (10, 12). For this reason, those inconsistencies identified by manual review were modified.

Research on RSV has shown variability in recent years in its focus on vaccines, antivirals or antibodies in the absence of lines that have demonstrated their efficacy. The clarification of the protection of newborns with the vaccination of pregnant women within clinical trials could modify the research in the coming years. Similarly, the development of new mechanisms of action (5), as well as the identification of vulnerable populations that may benefit more from the development of vaccines or drugs against influenza or RSV, such as cancer, immunosuppressed or high-risk subjects of cardiovascular events, are areas of future research.

## Data availability statement

The datasets presented in this study can be found in online repositories. The names of the repository/repositories and accession number(s) can be found at: clinicaltrials.gov.

## Author contributions

DL, MM-O, and EC: conceptualization. DL: data curation, software, validation, and writing—original draft. DL and MM-O: formal analysis, methodology, and visualization. DL, AG-R, AL, GM, MR-R, and MM-O: funding acquisition. DL, AG-R, AL, GM, and MR-R: investigation. EC and MM-O: supervision. DL, AG-R, AL, GM, MR-R, EC, and MM-O: writing—review and editing. All authors contributed to the article and approved the submitted version.
